# Supplementary figures and images for: Protein intrinsically disordered regions have a non-random, modular architecture
Source: Bioinformatics. 2023 Dec 1;39(12):btad732. doi: 10.1093/bioinformatics/btad732 (PMC10719218; doi:10.1093/bioinformatics/btad732)

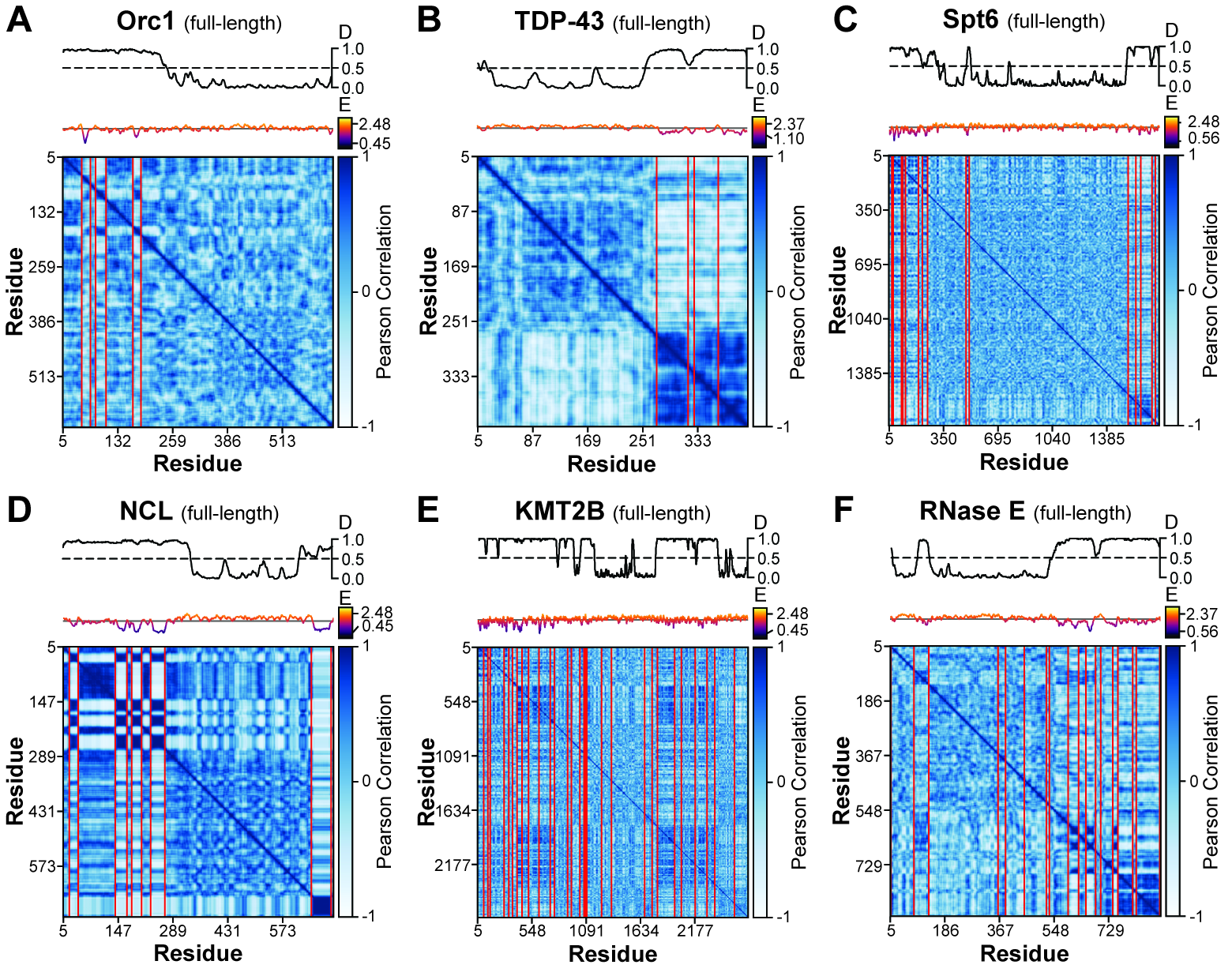

Supplement: btad732_Supplementary_Data [file btad732_supplementary_data.zip › Supplementary Fig. S2.tif]
